# Factors associated with change over time in quality of life of people with dementia: longitudinal analyses from the MODEM cohort study

**DOI:** 10.1186/s12877-022-03142-z

**Published:** 2022-05-31

**Authors:** Derek King, Nicolas Farina, Clare Burgon, Yvonne Feeney, Sharne Berwald, Elizabeth Bustard, Laura Gallaher, Ruth Habibi, Raphael Wittenberg, Adelina Comas-Herrera, Martin Knapp, Sube Banerjee

**Affiliations:** 1 Care Policy and Evaluation Centre, London School of Economics and Political Science, London, WC2A 2AE UK; 2 Centre for Dementia Studies, Brighton and Sussex Medical School, Brighton, BN1 9QY UK; 3 Faculty of Medicine & Health Sciences, University of Nottingham, Nottingham, NG7 2UH UK; 4 Faculty of Health, University of Plymouth, Plymouth, PL4 8AA UK

**Keywords:** Quality of life, Dementia, Alzheimer’s disease, Wellbeing, Health, Cognitive impairment, Caregiver, Longitudinal studies

## Abstract

**Background:**

Research to date offers mixed evidence about the relationship between quality of life and severity of cognitive impairment in people with dementia. We aimed to investigate longitudinal changes in patient- and proxy-rated health-related quality of life (HRQL) by severity of dementia and explore factors associated with changes in HRQL over a one-year period. We used data from the MODEM longitudinal cohort study which recruited dyads of persons with clinically diagnosed dementia and their principal carer and interviewed them face-to-face at baseline and again 1 year later.

**Methods:**

Quota sampling was used to generate balanced numbers (target *n* = 100 for each severity level) of people with mild cognitive impairment (20+ on the standardised Mini-Mental State Examination (sMMSE)), moderate cognitive impairment (score 10 to 19), and severe cognitive impairment (score 0 to 9). Persons with dementia without an identifiable family carer or other informant (e.g., a formal/professional/paid carer) were excluded from the study. Participants answered a series of questions measuring their HRQL: DEMQOL, DEMQOL-proxy, EQ-5D-3 L, EQ-5D-3L proxy. Multiple regression models were built to understand the effects of baseline demographics and dementia symptoms (cognitive impairment, neuropsychiatric symptoms) on change in HRQL over 1 year.

**Results:**

Two hundred and forty-three dyads of people with clinically diagnosed dementia and carers completed baseline and follow-up interviews. Most measures of HRQL remaining relatively stable between time-points, but one index of HRQL, EQ-5D proxy, significantly declined. Depending on the HRQL measure, different factors were associated with change in HRQL. The only factor consistently associated with decline in HRQL (when compared to improvement) was having a diagnosis of a non-Alzheimer’s dementia.

**Conclusions:**

Deterioration in HRQL is not an inevitable part of the dementia journey. However, people with non-Alzheimer’s dementias may be more susceptible to HRQL decline. This may indicate that those with non-Alzheimer’s dementia may benefit from specific support focussed on maintaining their quality of life.

## Background

Dementia is a collection of progressive neurodegenerative disorders with some common symptoms but wide interpersonal differences in experience and impact. The extent and speed of onset of cognitive and functional impairment vary considerably, and neuropsychiatric symptoms such as agitation, aggression, depression and anxiety can arise unpredictably at any stage [1, 2]. The course of illness can last from 3 to 12 years [3], and most people living with dementia will have other long-term physical health problems [4]. Taken together, there is enormous heterogeneity in the lives of people with dementia, which will impact variably on their quality of life.

Intuitively, it is often assumed that, as the severity of dementia increases, so the health-related quality of life (HRQL) must decline. In fact, previous research offers mixed evidence about the relationship between quality of life and severity of cognitive impairment in people with dementia [5–7]. Some authors have found that greater severity of cognitive impairment was not associated with lower HRQL (e.g. [8, 9]). We aimed to collect high-quality data on quality of life and other domains from a sample of people with dementia and their carers (as proxy-reporters), and to explore patterns of change over a 1-year period. To ensure that the sample included a sufficient number of people with severe dementia, quota sampling was used to capture samples balanced in numbers across disease severity. In this way, we were able to investigate changes in HRQL over time as severity of dementia increased. Previous studies suggest that patient-rated HRQL remains fairly stable over time and the course of the disease, while proxy ratings of patient HRQL tend to be lower and decline over time and across disease stages [10–15]. However, many studies are based on small samples with limited variation and focus on people with mild or moderate dementia severity at baseline.

## Methods

### Study design and sample

The MODEM project is modelling how changes in the treatment and care of people with dementia in England, and support for their carers, might result in better outcomes and more efficient use of resources [16]. One component of the project is to get a better understanding of health-related quality of life and what factors affect it. We recruited dyads of persons with clinically diagnosed dementia, of whatever cause, interviewed them and their carers face-to-face interviews. The baseline interviews took place between June 2015 and October 2016 and a follow-up interview was conducted, if possible, after 1 year [9]. We used quota sampling to generate balanced numbers (target *n* = 100 for each level of severity) of people with mild cognitive impairment (20+ on the standardised Mini-Mental State Examination (sMMSE)) [17], moderate cognitive impairment (score 10 to 19), and severe cognitive impairment (score 0 to 9). Persons with dementia without an identifiable family carer or other informant (e.g., a formal/professional/paid carer) were excluded from the study.

Ethical approval was obtained from the Social Care Research Ethics Committee (15/IEC08/0005).

Recruitment took place in South East England. People with dementia were recruited from: memory assessment and other older people’s mental health services; the Join Dementia Research national electronic database (joindementiaresearch.nihr.ac.uk/); community groups; and care homes. The people with dementia were formally assessed by a research worker (RW) to assess capacity to consent to participate in the study. Informed consent was obtained from those persons with dementia with capacity to do so. If they lacked capacity to consent, a personal consultee (family member or friend) was identified to advise on whether the person with dementia should take part. Interviews of the person with dementia and their carer took place simultaneously, with pairs of RWs visiting them in their own household or other agreed location. Capacity to consent was formally evaluated again at the follow-up interview 1 year later.

### Measures

Quality of life is a multidimensional construct incorporating an “*individual’s perception of their position in life in the context of culture and value systems in which they live and in relation to their goals, expectations, standards, and concerns*” [18]. Health-related quality of life refers to that element of quality of life that is driven by health and illness. The US Centres for Disease Control define HRQL as “*An individual’s or group’s perceived physical and mental health over time*” [19]. A number of domains were measured in the study at baseline and 1 year later.

#### Health-related quality of life

The disease-specific instruments DEMQOL and DEMQOL-Proxy [20] and the generic HRQL instruments EQ-5D-3L and EQ-5D-3L Proxy [21] were used. As stipulated in its manual, only persons with mild or moderate dementia were asked to complete DEMQOL, while all carers were asked to complete DEMQOL-Proxy. Scores on DEMQOL can range from 28 to 112 and on DEMQOL-Proxy scores from 31 to 124. On both measures, higher scores indicate better HRQL. The three-level version of self-report EQ-5D was used to produce utility ratings after applying preference weights derived from a UK population [22]. As with DEMDOL, EQ-5D-3L data were not collected for those with severe dementia.

#### Cognition

Severity of cognitive impairment was rated using the sMMSE [17].

#### Activity limitation

Carers were asked to assess the level of functioning of the cared-for person using the Bristol Activities of Daily Living Scale (BADLS) [23]. The questionnaire asks respondents to rate the person with dementia’s ability to complete activities of daily living (ADLs), such as eating and bathing, and instrumental activities of daily living (IADLs), such as cooking and taking medication. For the analysis, four levels of disability were defined: no ADL or IADL needs, needs help with one or more IADLs but no ADLs, difficulty with one ADL and difficultly with two or more ADLs.

#### Neuropsychiatric symptoms in dementia

The Neuropsychiatric Inventory (NPI) [24] was used to obtain a carer report of the presence and severity of neuropsychiatric (i.e. behavioural) disturbances which can occur in dementia. NPI scores can range from 0 to 144 with higher scores representing more severe behavioural disturbance.

#### Carer burden

Carers participating in the MODEM study were also asked to complete measures pertaining to their own well-being. The Zarit Burden Interview captures the stresses experienced by carers of persons with dementia [25]. It consists of 22 questions, possible scores ranging from 0 to 88. Higher scores indicate greater carer burden.

#### Carer mental well-being

The mental health of carers was assessed using the 12-item General Health questionnaire (GHQ) [26], a tool to identify common psychiatric disorders, and the 12-item Short Form Health Survey (SF-12) [27]. Scores used in the analysis were derived using the GHQ scoring algorithm with higher scores indicating greater mental health difficulties disorder and the mental health composite score for SF-12, for which higher scores indicate better mental health.

#### Socio-demographic data

A range of socio-demographic information was collected in interviews. In the analysis we included the age and gender of the person with dementia, the type of dementia (Alzheimer’s disease versus other), their education level and whether they lived in an urban or rural area. Also included was the relationship of the carer to the person with dementia and whether or not the carer was co-resident.

### Analysis

The initial step in the analysis was to summarise all measures using descriptive statistics. We compared the subsample of dyads for whom we had data at both time-points with those for whom we only had baseline data. Follow-up interview did not take place due to refusal to participate or loss to follow-up, including cases where the person with dementia died during the study period. Statistical comparisons of ‘completers’ with each of the other two subgroups (‘lost-to-follow-up’ and ‘died’) used the t-test for continuous measures and chi-squared test for categorical measures. These tests allowed us to assess if attrition was associated with any socio-demographic characteristics.

Next, we calculated summary statistics for the HRQL outcomes (EQ-5D, EQ-5D Proxy, DEMQOL, and DEMQOL-Proxy) at baseline and follow-up, and for the difference over time (follow-up score minus baseline score).

Prior to conducting statistical modelling, we imputed missing values to avoid bias in the estimates. Data were imputed for the subsample who declined to participate in the follow-up interview or were lost to follow-up, but not in cases where the person with dementia died during the study period. Fewer than 10% of the sample declined to participate in the follow-up or were lost to follow-up. We assumed that the data were missing at random (that is, factors associated with missingness were observed in the data), but not missing completely at random (which we determined by the analysis comparing ‘completers’ and ‘non-completers’ described above). Multiple imputation was used as this is preferrable to complete-case analysis when data are assumed missing at random as complete-case analysis can result in biased and inefficient estimates [28]. The multiple imputation used the method of chained equations [29], in which missing values are derived from an appropriate distribution of the partially observed data. Twenty copies of the dataset were derived using this method. The modelling results combine the estimates derived from each copy, incorporating standard errors associated with the uncertainty resulting from estimation across the multiple copies.

Models were estimated for each of the HRQL outcomes reported by the person with dementia (EQ-5D and DEMQOL) and by the carer (EQ-5D Proxy and DEMQOL-Proxy).

Variables considered in the modelling were selected on the basis on existing evidence [6, 7, 30], and consensus within the experienced research team. Based on previous cross-sectional analyses [9], we hypothesised that cognitive impairment (as measured by sMMSE) would have a greater association with proxy-rated HRQL than self-reported HRQL. Of particular interest is the assessment of the clinical significance of any association found to be statistically significant.

The modelling sought to take advantage of data collected at two time-points to reduce potential endogeneity. That is, that the quality of life of the person with dementia may be determined by an unobserved variable that is also correlated with one of the explanatory variables considered; or that quality of life is co-determined with one or more of the dementia symptom variables included in our modelling. By estimating effects of outcome measures at baseline on outcome measures at follow-up we reduce the potential for bias caused by endogeneity. Models for each of the HRQL outcomes took this approach in the first instance (model 1).

It is also worth considering the impact of changes over time in health state and disability that occur contemporaneously with changes in HRQL. This is particularly the case in considering the relative strength of association of changes in cognitive function, physical disability and behaviour disturbance with changes in HRQL. A second model estimated these contemporaneous effects (model 2).

Linear regression models were estimated. Different approaches exist for modelling change scores in regression analysis. We used the regressor variable method here as recommended in cases where the dependent variable has an inherent persistence over time unless altered by some specific process [31]. We assumed this to be the case for HRQL. Thus, the dependent variable in each model was the quality of life (QoL) measure at follow-up. In model 1, the independent variables were: the QoL measure at baseline; age, gender, marital status, education level and residential setting (urban vs rural) of the person with dementia; whether or not the carer co-resided with the person with dementia; carer’s relationship to the person with dementia; carer’s Zarit burden, GHQ and SF-12 mental health scores; whether the person with dementia had Alzheimer’s disease or another type of dementia; and severity of their cognitive impairment, ADL/IADL limitation and behavioural disturbance (NPI score).

Because of the strong correlation between carer burden, GHQ and SF-12 mental health scores, these were run in separate models in turn, with the final model retaining the variable with the greatest statistical significance. The same procedure was followed for a second model (model 2), in which dummy variables indicating increases in severity of cognitive impairment, level of physical disability and behavioural disturbance were added. Dummy variables were assigned a value of 1 if an increase occurred between baseline and follow-up or 0 if levels reduced or stayed the same. All models were estimated with robust standard errors, that is, standard errors robust to the error term not having constant variance. The goodness of fit was assessed for each model by graphing the residuals of each model against its fitted values and the standardized normal probability plot.

To further interrogate the data, we conducted analysis of change over time in EQ-5D Proxy and DEMQOL-Proxy variables after categorizing the values. Categories were defined based on estimates in the literature of the minimum (clinically) important difference (MID) on each measure. For each we created three categories: improvement, remained the same, and deterioration in quality of life. On EQ-5D Proxy, Coretti et al. [32] reviewed studies that estimated the MID in EQ-5D across a range of conditions using UK preference weights: median estimate was 0.14. Using an instrument-defined health state transitions approach, Luo [33] estimated the minimally improvement difference as 0.08 for the UK. As a point between these two estimates, we defined values of 0.1 or more on EQ-5D Proxy as representing important improvement and deterioration as decreases of more than 0.1. On DEMQOL-Proxy, Smith et al. [20] observed MID statistics ranging from 2 to 6 points (on 100-point scale) when using anchor and distribution-based methods on data from dementia carers. Based on this study, improvement on DEMQOL-Proxy was defined as values increasing by 4 units; deterioration as decreases of more than 4 units. Each categorical variable was modelled as a dependent variable in multinomial regression models (improvement as the reference category) with the same covariates as in the ordinary least squares models. Modelling quality of life categorically allowed us to assess the association of covariates with MID on EQ-5D Proxy and DEMQOL-Proxy.

*P*-values below 0.05 in statistical models were deemed statistically significant. Analysis was performed using STATA 14.2 [34].

## Results

### Participants

At baseline, 307 dyads were interviewed. The sample consisted of 110 persons with dementia with mild cognitive impairment, 100 with moderate cognitive impairment and 97 with severe cognitive impairment. The baseline sample is further described elsewhere [9]. Follow-up interviews were completed with 243 dyads. Of the remaining dyads in the study at baseline, 26 were lost to follow-up and in 38 cases the person with dementia had died within the year.

Table 1 compares the sub-sample that completed follow-up with those lost to follow-up and those in which the person with dementia had died. A significantly greater proportion of those lost to follow-up were men as compared to those who completed the study and those who died. Deaths were more common among single persons with dementia, those in care homes and those with severe cognitive impairment at baseline.

**Table 1:** Demographics of persons with dementia (at baseline) – percentages unless otherwise stated

	Follow-up complete (*n* = 243)	Refusal or lost to follow-up (*n* = 26)	Died (*n* = 38)	*p*-value*
Age: mean (SD)	80.1 (8.6)	81.2 (7.2)	85.0 (7.3)	CvsR:0.566 CvsD:0.001
Gender				
Female	50.6	41.7	55.3	0.578
Marital status:				
Married/civil partner	72.2	75.0	55.3	0.092
Widowed	23.2	16.7	42.1	
Divorced	2.9	8.3	0.0	
Single	1.7	0.0	2.6	
Education:				
O-level/GCSE or below	52.1	65.2	59.5	0.495
AS/A-level	14.5	4.4	8.1	
Degree	33.3	30.4	32.4	
Type of accommodation				
Care home	18.7	12.5	42.1	0.003
Urban/rural				
Rural	39.3	33.3	34.2	0.731
Type of dementia				
Alzheimer’s disease	62.7	52.2	52.6	0.346
sMMSE Total score (baseline; 0–30): mean (SD)	15.9 (9.1)	14.8 (8.9)	8.6 (6.8)	CvsR:0.585
				CvsD:0.001
NPI Total Score (baseline; 1–144): mean (SD) **↓**	18.8 (14.9)	17.0 (12.2)	19.8 (17.8)	CvsR:0.425
				CvsD:0.627
EQ-5D (baseline; 0–1): mean (SD)	0.80 (0.23)	0.83 (0.18)	0.90 (0.12)	CvsR:0.631
				CvsD:0.053
EQ-5D proxy (baseline; 0–1): mean (SD)	0.53 (0.33)	0.60 (0.33)	0.42 (0.30)	CvsR:0.190
				CvsD:0.051
DEMQOL (baseline; 28–112): mean (SD)	91.6 (13.1)	92.2 (11.0)	92.6 (13.2)	CvsR:0.864
				CvsD:0.747
DEMQOL proxy (baseline; 31–124): mean (SD)	95.3 (13.5)	88.9 (12.8)	98.5 (15.2)	CvsR:0.051
				CvsD:0.199

*Comparing follow-up complete cases, refusals/lost to follow-up and deaths. Between group comparison *p*-values reported (CvsR = Follow-up complete vs Refusal; CvsD = Follow-up complete vs Died)

Table 2 presents the distribution of HRQL scores at baseline and follow-up and the distribution of the change in scores from baseline to follow-up. The self-reported EQ-5D was completed by only a few persons with severe cognitive impairment and persons with severe cognitive impairment were not asked to complete DEMQOL as per the instrument’s manual. Thus, the self-reported values represent the subsample with mild or moderate cognitive impairment. Minimal change was observed in these values.

**Table 2:** Distribution of HRQL measures at baseline and follow-up, and change over time (completers only)

	Baseline (T1)		Follow-up (T2)		Difference (T2-T1)				
	Mean	SD	Mean	SD	Mean	SD	95%CI	Min	Max
EQ-5D (*n*=153)^a^	0.82	0.22	0.82	0.20	0.00030	0.21	-0.033, 0.034	-0.81	0.21
EQ-5D proxy (*n*=225)^a^	0.53	0.33	0.47	0.35	-0.062	0.32	-0.10, -0.020	-0.96	1.01
DEMQOL (*n*=140)^b^	92.0	13.0	92.0	12.9	0.05	7.7	-1.2, 1.3	-21.0	23.0
DEMQOL proxy (*n*=241)^c^	95.2	13.6	97.8	13.2	2.6	10.2	1.3, 3.9	-36.0	36.0

*SD* Standard Deviation, *CI* Confidence Interval

^a^Scale from   -0.59 to 1.0

^b^Scale from 28 to 112

^c^Scale from 31 to 124

There was divergence in the results observed for change over time in proxy-reported HRQL. Mean EQ-5D Proxy values decreased statistically significantly between baseline and follow-up from 0.53 to 0.47. This contrasted with those of the dementia-specific HRQL measure DEMQOL-Proxy. The mean DEMQOL-Proxy values were statistically significantly higher at follow-up.

The results of linear regression models of follow-up EQ-5D and EQ-5D Proxy values are presented in Table 3. In both Model 1 (baseline covariates only) and Model 2 (baseline covariates plus dummy variables of change in cognitive impairment, disability and behavioural disturbance), self-reported EQ-5D at follow-up was significantly associated only with baseline EQ-5D and the constant term. In the EQ-5D Proxy Model 1, in addition to the association with baseline EQ-5D, severe cognitive impairment and difficulty with two or more ADLs were associated with lower HRQL of the person with dementia as rated by their carer. In Model 2, these baseline effects remained and an increase in cognitive impairment was also associated with lower HRQL at follow-up.

**Table 3:** Multiple regression models with follow-up EQ-5D and EQ-5D proxy values as dependent variables

	EQ-5D (*n* = 195) *Model 1*	EQ-5D (*n* = 195) *Model 2*	EQ-5D proxy (*n* = 269) *Model 1*	EQ-5D proxy (*n* = 269) *Model 2*
EQ-5D/EQ-5D proxy at baseline	0.35 **	0.34 **	0.26 **	0.27 **
Age	−0.0013	− 0.0014	− 0.0032	− 0.0038
Gender				
Female	–	–	–	
Male	−0.037	− 0.038	− 0.034	− 0.025
Education level				
Below A-level or equivalent	–	–	–	
AS/A-level	0.030	0.028	0.0049	0.013
Degree or higher	−0.031	− 0.032	0.024	0.031
Co-resident				
No	–	–	–	
Yes	0.076	0.077	−0.0061	0.0078
Setting				
Rural	–	–	–	
Urban	0.014	0.015	0.050	0.051
Relationship of carer				
Spouse	–	–	–	
Son/daughter (incl. in-law)	0.011	0.012	− 0.074	− 0.062
Other	0.030	0.032	−0.063	−0.049
Carer (Zarit) burden score	0.0022	0.0023	0.00065	0.00048
Carer GHQ score				
Carer SF-12 Mental health score				
Alzheimer’s disease				
No	–	–	–	–
Yes	0.037	0.038	0.0013	−0.0015
Severity of cognitive impairment				
Mild	–	–	–	
Moderate	0.041	0.041	−0.044	− 0.042
Severe			−0.16 *	−0.20 **
Level of disability				
No ADL or IADL limitations	–	–	–	
Needs help with 1+ IADL	−0.016	− 0.017	− 0.016	0.012
Difficulty with 1 ADL	−0.072	− 0.073	− 0.090	−0.067
Difficulty with 2+ ADLs	−0.051	−0.046	− 0.25 **	−0.20 **
Level of behavioural disturbance (NPI score)	−0.00024	−0.00031	− 0.0022	−0.0028
Increase in severity of cognitive impairment		0.0024		−0.11 *
Increase in level of disability		0.014		−0.020
Increase in level of behavioural disturbance		−0.0043		−0.057
Constant	0.51 **	0.51 **	0.82 **	0.89 **

Values in table are linear regression beta coefficients

*p < 0.05; ** *p* < 0.01

Table 4 presents the regression results for the models of DEMQOL and DEMQOL-Proxy values at follow-up. As with the models of EQ-5D, self-reported, dementia-specific HRQL at follow-up was not associated with any of the covariates apart from the baseline score. Aside from the association with baseline DEMQOL-Proxy score, carers of persons with a diagnosis of Alzheimer’s disease rated HRQL more highly than did carers of persons with other types of dementia. Additionally, an increase in the level of behavioural disturbance in the person with dementia was associated with lower proxy-reported quality of life at follow up.

**Table 4:** Multiple regression model with follow-up DEMQOL, DEMQOL proxy values as dependent variables

	DEMQOL (*n* = 195) Model 1	DEMQOL (*n* = 195) Model 2	DEMQOL proxy (*n* = 269) Model 1	DEMQOL proxy (*n* = 269) Model 2
DEMQOL/DEMQOL proxy at baseline	0.65 **	0.65 **	0.63 **	0.63 **
Age	0.030	0.016	0.065	0.044
Gender				
Female	–	–	–	–
Male	0.35	0.30	−1.12	−1.17
Education level				
Below A-level or equivalent	–	–	–	–
AS/A-level	−0.61	−0.49	−2.29	−2.42
Degree or higher	1.52	1.45	−2.08	− 1.85
Co-resident				
No	–	–	–	–
Yes	1.17	1.21	−1.50	−0.95
Setting				
Rural	–	–	–	–
Urban	0.56	0.68	−0.48	− 0.32
Relationship of carer				
Spouse	–	–	–	–
Son/daughter (include in-law)	2.65	2.64	−3.64	−3.16
Other	3.90	4.11	−1.42	−1.00
Carer burden (Zarit Burden score)			−0.031	−0.025
Carer GHQ score				
Carer SF-12 Mental health score	− 0.031	− 0.038		
Alzheimer’s disease				
No	–	–	–	–
Yes	0.13	0.29	3.44 *	3.38 *
Severity of cognitive impairment				
Mild	–	–	–	–
Moderate	1.70	1.58	1.55	1.80
Severe			3.87	4.11
Level of disability				
No ADL or IADL limitations	–	–	–	–
Needs help with 1+ IADL	−2.08	−1.90	−1.55	−1.42
Difficulty with 1 ADL	−1.83	− 1.59	1.71	1.39
Difficulty with 2+ ADLs	−1.83	− 0.66	0.56	0.94
Level of behavioural disturbance (NPI score)	−0.065	− 0.060	− 0.053	− 0.12
Increase in severity of cognitive impairment		−1.49		0.68
Increase in level of disability		1.33		0.71
Increase in level of behavioural disturbance		0.24		−4.32 **
Constant	30.72	31.91	34.71 **	38.02 **

Values in table are linear regression beta coefficients

*p < 0.05; ** *p* < 0.01

Modelling change in EQ-5D Proxy values as a categorical variable, the characteristics associated with poorer HRQL compared to improved HRQL were: lower EQ-5D value at baseline, severe cognitive impairment, difficulty with two or more ADLs, and greater behavioural disturbance (Table 5). Characteristics associated with a deterioration in HRQL on DEMQOL-Proxy compared to an improvement were: lower DEMQOL-Proxy value at baseline, having a type of dementia other than Alzheimer’s disease, and an increase in the level of behavioural disturbance (Table 6).

**Table 5:** Multiple regression model of change in HRQL as measured on EQ-5D proxy as dependent variable

	EQ-5D proxy (*n* = 269)		EQ-5D proxy (*n* = 269)	
	Poorer HRQL… compared to improvement	Stable HRQL… compared to improvement	Poorer HRQL… compared to improvement	Stable HRQL… compared to improvement
EQ5D proxy at baseline	8.03 **	4.51 **	8.14 **	4.55 **
Age	0.032	0.017	0.037	0.023
Gender				
Female	–	–	–	–
Male	0.23	0.47	0.16	−0.47
Education level				
Below A-level or equivalent	–	–	–	–
AS/A-level	−0.35	−0.032	− 0.39	−0.023
Degree or higher	−0.53	−0.60	− 0.61	−0.55
Co-resident				
No	–	–	–	–
Yes	−0.038	−0.86	− 0.13	−0.86
Setting				
Rural	–	–	–	–
Urban	−0.40	−0.13	− 0.43	−0.18
Relationship of carer				
Spouse	–	–	–	–
Son/daughter (incl. in-law)	0.42	−0.14	0.34	−0.16
Other	−0.26	−1.13	−0.42	−1.20
Carer burden (Zarit Burden score)	−0.014	−0.031 *	− 0.015	−0.033 *
Alzheimer’s disease				
No	–	–	–	–
Yes	−0.44	0.12	−0.44 *	0.082
Severity of cognitive impairment				
Mild	–	–	–	–
Moderate	0.62	0.68	0.60	0.70
Severe	1.63 *	1.61 *	1.87 *	1.78 *
Level of disability				
No ADLs or IADLs	–	–	–	–
Needs help with 1+ IADL	−0.012	−0.95	−0.17	−0.97
Difficulty with 1 ADL	0.23	−0.14	0.13	−0.16
Difficulty with 2+ ADLs	2.29 *	0.56	2.20 *	0.22
Level of behavioural disturbance (NPI score)	0.037 *	0.027	0.043 *	0.029
Increase in severity of cognitive impairment			0.55	0.23
Increase in level of disability			0.23	−0.68
Increase in level of behavioural disturbance			0.45	0.029
Constant	−7.65 *	−2.81	−8.39 **	−3.05

Values in table are multinomial regression beta coefficients

*p < 0.05; **p < 0.01

**Table 6:** Multiple regression model of change in dementia quality of life as measured on DEMQOL proxy

	DEMQOL proxy (*n* = 269)		DEMQOL proxy (*n* = 269)	
	Poorer HRQL… compared to improvement	Stable HRQL… compared to improvement	Poorer HRQL… compared to improvement	Stable HRQL… compared to improvement
DEMQOL proxy at baseline	0.077 **	0.070 **	0.080 **	0.069 **
Age	−0.029	0.022	−0.030	0.024
Gender				
Female	–	–	–	–
Male	0.52	−0.41	0.58	−0.44
Education level				
Below A-level or equivalent	–	–	–	–
AS/A-level	0.37	−0.60	0.44	−0.62
Degree or higher	0.49	0.32	0.43	0.27
Co-resident				
No	–	–	–	–
Yes	0.88	0.91	0.75	0.86
Setting				
Rural	–	–	–	–
Urban	0.40	0.21	0.43	0.22
Relationship of carer				
Spouse	–	–	–	–
Son/daughter (incl. in-law)	1.30	0.22	1.23	0.16
Other	0.50	−0.59	0.51	−0.70
Carer burden (Zarit Burden score)	−0.0065	− 0.025	−0.0083	− 0.026
Alzheimer’s disease				
No	–	–	–	–
Yes	−0.95 *	−1.28 **	−0.91 *	−1.27 **
Severity of cognitive impairment				
Mild	–	–	–	–
Moderate	0.27	−0.60	0.22	−0.62
Severe	−0.65	−0.87	− 0.98	−0.81
Level of disability				
No ADLs or IADLs	–	–	–	–
Needs help with 1+ IADL	0.47	0.48	0.46	0.40
Difficulty with 1 ADL	−0.43	−0.38	−0.37	− 0.42
Difficulty with 2+ ADLs	−0.32	− 0.025	−0.13	− 0.075
Level of behavioural disturbance (NPI score)	0.017	0.016	0.035	0.020
Increase in severity of cognitive impairment			−0.71	0.17
Increase in level of disability			0.29	0.17
Increase in level of behavioural disturbance			1.12 *	0.32
Constant	−6.57 *	−7.54 **	−7.43 **	−7.71 **

Values in table are multinomial regression beta coefficients

*p < 0.05; **p < 0.01

## Discussion

There are around 690,000 people with dementia in England [35] and 50 million worldwide [36]. Dementia has profound impacts on the individuals with dementia themselves, their families, and society in general. The syndrome of dementia can be caused by a number of different disorders, with Alzheimer’s disease the most common [37], but up to a third may be caused by other conditions including vascular dementia, Lewy Body dementia, frontotemporal dementia and mixed dementias.

The impact of dementia on quality of life can be profound. In this study, we described patterns of quality of life, using both self- and proxy-rated measures, over a 12-month period, and explored factors associated with changes in HRQL. We used careful quota-sampling to include sufficient numbers of people with different levels of dementia severity.

### Strengths and limitations

A limitation of this study is that we have only two time-points, 12 months apart. Richer information would have been available if we had been able to measure intermediate time-points and to follow the sample for longer than 12 months. Two time-points limit us to assuming a linear relationship, which may not be the best fit for actual effects on quality of life over time. Studies with longer follow-up periods may see greater loss to follow up; we were able to include 69% of the original sample in our longitudinal analysis, which is comparable or better than similar longitudinal studies [12, 14, 15, 38].

Another limitation is that self-report data, which should always have primacy in quality of life assessment, were only available for those with mild and moderate severity of dementia because of the cognitive impairment that is a fundamental element of the disorder. The sample also reflects a somewhat homogenous group of people with dementia, who notably were well-educated, White British (see [9]) and recruited from a single geographic region of England.

Strengths of the approach taken here were that: we were able to follow up equal numbers of people with mild, moderate, and severe cognitive impairment, including people in care homes; we compared disease-specific and generic measures head-to-head in self- and proxy-report formats; and we achieved a high level of follow-up (90%; 243/269) of those who survived (death rate = 12.4%; 38/307).

### Interpretations

These findings provide insights into the natural history of HRQL in dementia and the effect of severity of cognitive impairment on change in HRQL. Focussing on the proxy measures which cover the whole range of dementia severity, it is striking that contradictory results were observed for change over time in quality of life from generic and disease-specific measures of HRQL.

Mean scores on the (generic) EQ-5D proxy decreased statistically significantly between baseline and follow-up from 0.53 to 0.47 (95%CI − 0.10 to − 0.02) while mean scores on the (disease-specific) DEMQOL-Proxy increased from 95.2 to 97.8 (95%CI 1.3 to 3.9). Whilst there was a statistically significant decline in the EQ-5D Proxy, and an increase in the DEMQOL-Proxy, it does not mean that change is clinically meaningful. It is important to recognise that on average HRQL remained relatively stable, which supports findings reported elsewhere (e.g. [39]). There have been a variety of methods proposed to determine what is clinically meaningful change [40]. The present sample did not display such a change, irrespective of HRQL outcome, either based on the estimates of MID we employed, or on the basis that a clinically meaningful change is greater than half a standard deviation of the total score [41]. It should be noted that at an individual level there might be a degree of variability [42].

In an earlier paper, we looked at cross-sectional data from the MODEM cohort to explore factors associated with quality of life using an a priori model, with cognitive impairment being a key variable [9]. Unlike the new analyses presented here, the cross-sectional data did not show systematic differences between generic and disease-specific measures when reported by the person with dementia. Proxy-report measures were associated with cognitive impairment. This has also been observed in cross-sectional studies [10, 13, 43] and other longitudinal studies [11–15]. The present findings, do however support observations from the DADE2 study in which a significant decline in the EQ-5D Proxy occurred alongside a significant improvement in the DEMQOL-Proxy scores over 18-months [38].

One possible explanation of our findings is that the EQ-5D Proxy is subject to greater error in dementia samples than the DEMQOL-Proxy. By definition, generic measures are designed to work for all disorders; they focus on a core set of physical and mental functions and therefore (potentially) miss elements that are of particular salience in specific disorders. Given the considerable complexity of the syndrome of dementia, where simple measures of function are poor indicators of HRQL, the generic proxy measure may be measuring the inevitable increase in activity limitation that is inherent in dementia, rather than HRQL where no such direct relationship with severity exists [5]. The DEMQOL questionnaire was developed from a theoretical bases, with people with dementia as the respondent so it is more likely to capture what they feel is important to their HRQL [20].

Another possible explanation may stem from the framing of some DEMQOL-Proxy questions. The DEMQOL-Proxy (and DEMQOL) benefit from capturing how worried people with dementia are about various aspects of their lives, and clearly distinguishes itself from other measures which are more focussed on functional ability (e.g., QoL-AD). This means that other HRQL measures will inevitably decline over time as it is tied with functional performance. The DEMQOL-Proxy is likely to be less susceptible to this but might introduce different sources of bias. For example, insight into cognitive impairment may dictate the extent to which someone is worried. Certainly, awareness of memory function is a strong predictor of HRQL [44]. Whilst ignoring the dementia and living in the moment may act as a coping mechanism to preserve quality of life [45]. This would be reflected in improvement in DEMQOL-Proxy scores over time, as observed.

The consistency in findings between model 1 and model 2 suggests that significant effects are robust and are not enhanced by a more strict temporal interpretation of their relationship to quality of life in persons with dementia. The one exception is the significance of change in the level of behavioural disturbance on DEMQOL-proxy values and the non-significance of baseline level of behavioural disturbance on this outcome measure.

Interestingly, EQ-5D proxy decline appeared to be driven by measures of disease severity at baseline (cognition and ADLs). Such associations were not observed in any other outcome measure. However, change in cognitive impairment over time was only significantly associated with the EQ-5D proxy scores when treated as a continuous outcome. This association did not exist when the EQ-5D proxy deterioration or stability was compared to improvement, indicating disease progression is likely to account for a small amount of variance in the outcome. Therefore, for at least the EQ-5D proxy outcome, the absolute stage of the disease is a more important determinant of HRQL than changes in cognitive performance over a 12-month period.

There was some variation in the factors associated with poorer HRQL, depending on the model and measure of HRQL. One recurring factor associated with the difference between those whose HRQL improved and those whose HRQL deteriorated was dementia diagnosis. In cross-sectional baseline analyses from the IDEAL project, people with non-Alzheimer’s disease dementia scored lower on measures spanning quality of life (including HRQL), life satisfaction and wellbeing [46]. That study noted that people with Parkinson’s disease dementia and Lewy body dementia often had the worst outcomes, attributing the association to symptoms such as fatigue, hallucinations or other comorbidities. This supports further the need to better understand why certain subtypes of dementia are more likely to experience a worsening of quality of life in general.

## Conclusion

The complexity of dementia has led to acknowledgement that broad measures of overall impact and outcome are needed to develop, deliver and monitor treatment and care that enables people to live well with the condition. This study highlights that worsening HRQL is not an inevitable part of the dementia journey. However, the choice of outcome measure has a pivotal role in the associations found. Generic measures of HRQL are essential in the evaluation of interventions, particularly to inform resource allocation decisions across different health problems, but our findings clearly demonstrate the value of also using disease-specific measures. Irrespective of the method used, however, increasing cognitive impairment does not appear to be a key driver of poorer HRQL over time. Our finding that people with non-Alzheimer’s dementia appear to have a worse prognosis in HRQL terms warrants further exploration. This may indicate that those with non-Alzheimer’s dementia may benefit from specific support focussed on maintaining their quality of life.

## Data Availability

Data will be made available upon reasonable request to the corresponding author.

## Abbreviations

HRQL: Health-related quality of life
sMMSE: Standardised Mini-Mental State Examination
RW: Research worker
ADL: Activities of Daily Living
IADL: Instrumental Activities of Daily Living
NPI: Neuropsychiatric Inventory
GHQ: General Health Questionnaire
SF-12: Short Form Health Survey

## References

[1] Kazui H, Yoshiyama K, Kanemoto H, Suzuki Y, Sato S, Hashimoto M (2016). Differences of behavioral and psychological symptoms of dementia in disease severity in four major dementias. PLoS One.

[2] Landes AM, Sperry SD, Strauss ME (2005). Prevalence of apathy, dysphoria, and depression in relation to dementia severity in Alzheimer’s disease. J Neuropsychiatry Clin Neurosci.

[3] Todd S, Barr S, Roberts M, Passmore AP (2013). Survival in dementia and predictors of mortality: a review. Int J Geriatr Psychiatry.

[4] Kurrle S, Brodaty H, Hogarth R (2012). Physical comorbidities of dementia.

[5] Banerjee S, Samsi K, Petrie CD, Alvir J, Treglia M, Schwam EM (2009). What do we know about quality of life in dementia? A review of the emerging evidence on the predictive and explanatory value of disease specific measures of health related quality of life in people with dementia. Int J Geriatr Psychiatry.

[6] Jing W, Willis R, Feng Z (2016). Factors influencing quality of life of elderly people with dementia and care implications: a systematic review. Arch Gerontol Geriatr.

[7] Martyr A, Nelis SM, Quinn C, Wu Y-T, Lamont RA, Henderson C (2018). Living well with dementia: a systematic review and correlational meta-analysis of factors associated with quality of life, well-being and life satisfaction in people with dementia. Psychol Med.

[8] Banerjee S, Smith SC, Lamping DL, Harwood RH, Foley B, Smith P (2006). Quality of life in dementia: more than just cognition. An analysis of associations with quality of life in dementia. J Neurol Neurosurg Psychiatry.

[9] Farina N, King D, Burgon C, Berwald S, Bustard E, Feeney Y (2020). Disease severity accounts for minimal variance of quality of life in people with dementia and their carers: analyses of cross-sectional data from the MODEM study. BMC Geriatr.

[10] Bosboom PR, Alfonso H, Eaton J, Almeida OP (2012). Quality of life in Alzheimer’s disease: different factors associated with complementary ratings by patients and family carers. Int Psychogeriatr.

[11] Bosboom PR, Alfonso H, Almeida OP (2013). Determining the predictors of change in quality of life self-ratings and carer-ratings for community-dwelling people with Alzheimer disease. Alzheimer Dis Assoc Disord.

[12] Clare L, Woods RT, Nelis SM, Martyr A, Marková IS, Roth I (2014). Trajectories of quality of life in early-stage dementia: individual variations and predictors of change. Int J Geriatr Psychiatry.

[13] Huang H-L, Weng L-C, Tsai Y-H, Chiu Y-CY, Chen K-H, Huang C-C (2015). Predictors of self- and caregiver-rated quality of life for people with dementia living in the community and in nursing homes in northern Taiwan. Int Psychogeriatr.

[14] Missotten P, Ylieff M, Notte DD, Paquay L, Lepeleire JD, Buntinx F (2007). Quality of life in dementia: a 2-year follow-up study. Int J Geriatr Psychiatry.

[15] Tatsumi H, Nakaaki S, Torii K, Shinagawa Y, Watanabe N, Murata Y (2009). Neuropsychiatric symptoms predict change in quality of life of Alzheimer disease patients: a two-year follow-up study. Psychiatry Clin Neurosci.

[16] Comas-Herrera A, Knapp M, Wittenberg R, Banerjee S, Bowling A, Grundy E (2017). MODEM: a comprehensive approach to modelling outcome and costs impacts of interventions for dementia. Protocol paper. BMC Health Serv Res.

[17] Molloy DW, Alemayehu E, Roberts R (1991). Reliability of a standardized Mini-mental state examination compared with the traditional Mini-mental state examination. Am J Psychiatry.

[18] The WHOQOL Group (1995). The World Health Organization quality of life assessment (WHOQOL): position paper from the World Health Organization. Soc Sci Med.

[19] Centers for Disease Control and Prevention (2000). Measuring healthy days: population assessment of health-related quality of life.

[20] Smith SC, Lamping DL, Banerjee S, Harwood R, Foley B, Smith P (2005). Measurement of health-related quality of life for people with dementia: Development of a new instrument (DEMQOL) and an evaluation of current methodology. Health Technol Assess.

[21] EuroQol Group (1990). EuroQol—A new facility for the measurement of health-related quality of life. Health Policy.

[22] Dolan P (1997). Modeling valuations for EuroQol health states. Med Care.

[23] Bucks RS, Ashworth DL, Wilcock GK, Siegfried K (1996). Assessment of activities of daily living in dementia: development of the Bristol activities of daily living scale. Age Ageing.

[24] Cummings JL, Mega M, Gray K, Rosenberg-Thompson S, Carusi DA, Gornbein J (1994). The neuropsychiatric inventory: comprehensive assessment of psychopathology in dementia. Neurology.

[25] Zarit SH, Reever KE, Bach-Peterson J (1980). Relatives of the impaired elderly: correlates of feelings of burden. Gerontologist.

[26] Goldberg D, Williams P (2000). General health questionnaire (GHQ).

[27] Ware JE, Kosinski M, Keller SD (1996). A 12-item short-form health survey: construction of scales and preliminary tests of reliability and validity. Med Care.

[28] Sterne JAC, White IR, Carlin JB, Spratt M, Royston P, Kenward MG (2009). Multiple imputation for missing data in epidemiological and clinical research: potential and pitfalls. Br Med J.

[29] Van Buuren S (2007). Multiple imputation of discrete and continuous data by fully conditional specification. Stat Methods Med Res.

[30] Farina N, Page TE, Daley S, Brown A, Bowling A, Basset T (2017). Factors associated with the quality of life of family carers of people with dementia: a systematic review. Alzheimers Dement.

[31] Allison PD (1990). Change scores as dependent variables in regression analysis. Sociol Methodol.

[32] Coretti S, Ruggeri M, McNamee P (2014). The minimum clinically important difference for EQ-5D index: a critical review. Expert Rev Pharmacoecon Outcomes Res.

[33] Luo N, Johnson JA, Coons SJ (2010). Using instrument-defined health state transitions to estimate minimally important differences for four preference-based health-related quality of life instruments. Med Care.

[34] StataCorp LP (2015). Stata/SE 14.2 for windows [64-bit x86–64].

[35] Wittenberg R, Knapp M, Hu B, Comas-Herrera A, King D, Rehill A (2019). The cost of dementia in England. Int J Geriatr Psychiatry.

[36] World Health Organization (2019). Dementia.

[37] Goodman RA, Lochner KA, Thambisetty M, Wingo T, Posner SF, Ling SM (2017). Prevalence of dementia subtypes in U.S. Medicare fee-for-service beneficiaries, 2011-2013. Alzheimers Dement.

[38] Trigg R, Jones RW, Knapp M, King D, Lacey LA (2015). The relationship between changes in quality of life outcomes and progression of Alzheimer’s disease: results from the dependence in AD in England 2 longitudinal study. Int J Geriatr Psychiatry.

[39] Laybourne A, Livingston G, Cousins S, Rapaport P, Lambe K, Frenais FL (2019). Carer coping and resident agitation as predictors of quality of life in care home residents living with dementia: managing agitation and raising quality of life (MARQUE) English national care home prospective cohort study. Int J Geriatr Psychiatry.

[40] Crosby RD, Kolotkin RL, Williams GR (2003). Defining clinically meaningful change in health-related quality of life. J Clin Epidemiol.

[41] Norman GR, Sloan JA, Wyrwich KW (2003). Interpretation of changes in health-related quality of life: the remarkable universality of half a standard deviation. Med Care.

[42] Yu H, Gao C, Zhang Y, He R, Zhou L, Liang R (2017). Trajectories of health-related quality of life during the natural history of dementia: a six-wave longitudinal study. Int J Geriatr Psychiatry.

[43] Akpınar Söylemez B, Küçükgüçlü Ö, Akyol MA, Işık AT (2020). Quality of life and factors affecting it in patients with Alzheimer’s disease: a cross-sectional study. Health Qual Life Outcomes.

[44] Trigg R, Watts S, Jones R, Tod A (2011). Predictors of quality of life ratings from persons with dementia: the role of insight. Int J Geriatr Psychiatry.

[45] Thorsen K, Dourado M, Johannessen A (2020). Awareness of dementia and coping to preserve quality of life: a five-year longitudinal narrative study. Int J Qual Stud Health Well Being.

[46] Wu Y-T, Clare L, Hindle JV, Nelis SM, Martyr A, Matthews FE (2018). Dementia subtype and living well: results from the improving the experience of dementia and enhancing active life (IDEAL) study. BMC Med.

